# Rebutting Oberhuber- Again

**Published:** 2015

**Authors:** Michael N Marsh, Matt W Johnson, Kamran Rostami

**Affiliations:** 1 *Department of Gastroenterology, Luton & Dunstable University Hospitals Trust, Luton, United Kingdom *; 2 *Wolfson College, University of Oxford, United Kingdom *; 3 *Department of Gastroenterology, Alexandra Hospital, Worcestershire, United Kingdom*

**To The Editor:**

In describing the ultrastructural features of "flat" celiac mucosae as observed by scanning electron microscopy (SEM), we clearly demonstrated that the surface terrain, although irregular, does not reveal "atrophic" and "partially degenerate" villi as Oberhuber (1) originally suggested and based on his examinations of vertical sections alone – and, in our opinion, incorrectly. Instead, we have described mosaic plateaux; and large basin-like wells into which open numerous crypt tubes and which are surrounded by concentric arrays of enterocytes – either flat or raised up into circumferential collars. We could not, on the other hand, recognise by SEM any of the subdivisions proposed earlier. Thus we cannot obviously agree with Oberhuber that we only showed the severest Marsh III lesion (2). There was, in fact, nothing else we could have demonstrated, and that is why we produced "cartoons" in order to point out the interpretational problems arising from Oberhuber's scheme. Again, we make the comment that the IIIa,b,c sub-classification had appeared in earlier work by Rostami and others (3).

 We are amazed by the statement that histopathologists spend time going through multiple "serial sections" in order to characterise (? routinely, we might ask) the precise Marsh III stage. If that is the necessary requirement, and obviously so critical to proper evaluation, why then is this approach not advocated and emphasised in their original paper? We thus merely view that proviso as a last-minute retrograde attempt to uphold their disorganised approach. Moreover, why are there no measurements in their paper which would surely aid discrimination? As we stated in our recent presentation in Prague, since there was no obviously consistent agreement in two papers offered to histopathologists on the critical issue of proper classification (1, 4) we concluded that perhaps Dickson and colleagues also did not have a definitive understanding of the necessary procedures required, either.

 In our experience, we have never come across any histopathologists who could extravagantly use their time to survey a series of serial sections (nor a routine hospital-based lab that could process all this extra material) in order to secure the right conclusion. The majority of pathologists are overwhelmed by the demanding task of reporting a massive daily load of new biopsies. Moreover, if Oberhuber's categories and controls are so tight as he claims, as well as routinely and consistently realised, how could an inexperienced histopathologist ever get it wrong? Their statement on this matter surprises us as well.

 Any new research should raise additional questions. The Oberhuber scheme offers no such added potential to understanding; it has never offered anything fruitful in terms either of further research, nor diagnosis, management, or prognosis. If it did, then there should have been further papers from other laboratories elaborating those issues. We know of no such papers.

 On the other hand, the Marsh Classification (5) has spurred much considerable work in defining the immunopathology of the evolving mucosal lesion. Far from our being "prey" to this concept – or even "disbelieving" in it, we firmly re-assert that this wider classification has admitted many, possibly several thousand, patients worldwide to a proper diagnosis during its 23 years of operation, thereby relieving the latter of their symptoms, repairing nutritional deficiencies, and possibly retarding the onset of lymphomatous change. That certainly cannot be said of Oberhuber.

 Oberhuber complains that the structural transition from Marsh II to Marsh III is incomplete. He obviously thinks that it should be possible to see "degenerate" or "atrophic" villi during that transitional phase. But that may well be an improbable and grossly misconceived viewpoint. For as we stated in our Discussion (2), and corroborated by appropriate data from earlier papers, we assert from the appearances seen on SEM that villi do NOT undergo complete attrition and wither, thus to be occasionally recognized by the Oberhuber scheme. If that were indeed the case, then they would surely be visible on the specimens we illustrated (Figures 1 and 2). Rather, it seems as though mosaic plateaux represent amalgamations of several adjacent villi, following their initial partial flattening and widening, as Padykula's study indicates (6). From that it follows that the mosaic platforms represent A conglomerate of villous, and not crypt, territory. On those grounds, Oberhuber's claims fail to provide any insights into that possibility because of his insistence that the mucosal surface must exhibit remnants of "degenerate" or "atrophic" villi. That can only amount to imaginative speculation.

 We must also remember that some other pathologists have rubber-stamped the Marsh I and II lesions as "non-specific" – a spurious claim rightly and peremptorily rejected by other histopathologists forming the core consensus of the Bucharest Working Party (7) and collectively reclassified by us as Microscopic Enteritis. That restores Marsh I and II to their rightful histopathological and diagnostic roles in respect of gluten sensitivity and to the evolution of the mucosal lesion. Finally, the definition of the Marsh 0 ("normal") appearance while making routine assessments very difficult, has nevertheless encouraged the view that this is another structural feature in the progress of the gluten-induced mucosal lesion (8) and thus forging new observations at this very early stage of pathology (9). There may even be more, if it can be shown that the recent, interesting findings of Korponay-Szabo and her colleagues are found to have further diagnostic usefulness (10).
